# The Performance Investigation of Smart Diagnosis for Bearings Using Mixed Chaotic Features with Fractional Order

**DOI:** 10.3390/s23083801

**Published:** 2023-04-07

**Authors:** Shih-Yu Li, Lap-Mou Tam, Shih-Ping Wu, Wei-Lin Tsai, Chia-Wen Hu, Li-Yang Cheng, Yu-Xuan Xu, Shyi-Chyi Cheng

**Affiliations:** 1 Graduate Institute of Manufacturing Technology, National Taipei University of Technology, Taipei 10608, Taiwan; 2 Institute for the Development and Quality, Macao 999078, China; 3 Department of Electromechanical Engineering, Faculty of Science and Technology, University of Macau, Macao 999078, China; 4 Master Program, Graduate Institute of Mechatronic Engineering, National Taipei University of Technology, Taipei 10608, Taiwan; 5 Department of Mechanical Engineering, National Taipei University of Technology, Taipei 10608, Taiwan; 6 Department of Computer Science and Engineering, National Taiwan Ocean University, Keelung City 202301, Taiwan

**Keywords:** ball bearings, fault detection, chaotic features, fractional order, extension theory

## Abstract

This article presents a performance investigation of a fault detection approach for bearings using different chaotic features with fractional order, where the five different chaotic features and three combinations are clearly described, and the detection achievement is organized. In the architecture of the method, a fractional order chaotic system is first applied to produce a chaotic map of the original vibration signal in the chaotic domain, where small changes in the signal with different bearing statuses might be present; then, a 3D feature map can be obtained. Second, five different features, combination methods, and corresponding extraction functions are introduced. In the third action, the correlation functions of extension theory used to construct the classical domain and joint fields are applied to further define the ranges belonging to different bearing statuses. Finally, testing data are fed into the detection system to verify the performance. The experimental results show that the proposed different chaotic features perform well in the detection of bearings with 7 and 21 mil diameters, and an average accuracy rate of 94.4% was achieved in all cases.

## 1. Introduction

The popularization of precision manufacturing has led to increasingly demanding accuracy requirements in machining and manufacturing processes to ensure the normal operation of machines and to avoid damage to ball bearings without warning, which may cause operational failure of the machine or public security accidents. Therefore, it is necessary to detect whether the ball bearing is in a normal state or damaged. Many problems can be avoided if repairs are carried out in time before the bearing fails. Therefore, the purpose of this research is to develop methods and tools to further detect whether a bearing is damaged or not [1,2].

Several studies have shown that physical signals, such as stator currents [3], acoustic signals [4], and vibration signals [5], can be utilized for data exploration in the diagnosis of bearing failures. Among these signals, vibration signals are the most widely used choice. In real industrial production operations, machine vibration signals are considered to be one of the most effective and abundant physical signal sources for detecting bearing failure. Therefore, analyzing vibration signals has become critically important, with wavelet transform and Fourier analysis being two of the most common techniques for studying vibration signals.

Fast Fourier transform (FFT) [6,7] and short-time Fourier transform (STFT) [8] are popular Fourier-based methods for detecting fault conditions in bearings. Despite their good recognition capabilities, the fixed transformation windows of FFT and STFT result in inflexible resolution, making it challenging to achieve satisfactory resolution in both the time and frequency domains simultaneously. This factor makes it difficult to achieve satisfactory time and frequency domain resolutions simultaneously. Discrete Fourier analysis (DFA) [9] is another method that can clearly define the frequencies, locations, and ranges of fault states, but it performs poorly when the data present nonlinearity and time–frequency variations.

Wavelet analysis [10,11] is commonly used in data mining for various applications, such as Integrated Resonance-Based Sparse Signal Decomposition (RSSD) [12] and Wavelet Transform (WT) for fault diagnosis. However, practical applications remain challenging due to the wide range of frequency characteristics in bearings, which makes it difficult to select an effective mother wavelet for wavelet analysis. Additionally, physical sensors are often needed for wavelet transformations, leading to high costs and error rates. In other studies, empirical modal decomposition (EMD) has been used for data analysis and feature extraction, while the Hilbert transformation (HT) has been used for bearing failure diagnosis. However, in practice, the broad frequency spectrum of bearings results in the processing of intricate signals, making it challenging to determine the appropriate mother wavelet for wavelet analysis.

An alternative method for fault diagnosis was proposed by Yau et al. [13,14]. This method utilizes the theory of chaotic synchronous error dynamics and fractal theory. The characteristics of fractal theory have been further used to intercept error features, resulting in effective fault diagnosis, although this method requires additional time and professional experience to adjust the fraction order. Based on previous research achievements in applying chaotic systems, a smart detection system with a hierarchical structure of different features was proposed; this method simplifies the system structure and improves the efficiency of detection.

Li and Gu et al. [15,16,17] proposed Chaotic Mapping systems associated with a classification method extension theory. These systems are effective in identifying bearing fault states, but the processing of dynamic error signals requires extra time, leading to delayed diagnosis. To solve this disadvantage, this study applies fractional order chaotic synchronization to extract fewer signal characteristics of ball bearings and uses five different proposed feature extraction methods. This method allows the current state of a ball bearing system to be easily identified into four main fault states using a chaotic system.

Artificial Intelligence (AI) [18] algorithms have been a subject of discussion recently, particularly the use of Artificial Neural Networks (ANNs) and the breakthroughs in computing power that have made Deep Neural Networks (DNNs) [19,20] and Deep Learning (DL) [21] possible. These algorithms have demonstrated success in solving complex nonlinear classification problems between factor and dependent variables. However, the use of DL is limited by the need for large amounts of training data, complex learning structures, and high-intensity computing capabilities, making real-time monitoring of bearing status challenging. In contrast, Machine Learning (ML) can effectively solve linear classification and prediction problems with fewer training data and lower computational resources, making real-time monitoring of bearing states more feasible. However, ML has limitations in accurately solving complex nonlinear classification problems.

In the middle of the 20th century, thanks to the invention of computers, people were able to simulate weather phenomena and analyze the results through numerical simulations. According to “Deterministic Nonperiodic Flow” [22], edited by Edward Norton Lorenz, who sought to explain weather phenomena with a chaotic system, when a nonlinear system of differential equations has particular parameters, the system will show the properties of chaos. Chaos theory has the following characteristics: 1. The system is extremely sensitive to initial conditions, and different initial values have wildly different results; 2. The system is non-periodic in the long term; and 3. The system is deterministic. Thus, when the initial value is determined, we can predict its behavior. The aforementioned three properties of chaotic systems have the ability to change the characteristics of the original signal. The processed signal then undergoes the feature extraction technique proposed in this paper, which can facilitate signal analysis. Two application fields for this method will be discussed. First, because the system is extremely sensitive to initial conditions and does not converge on a single point, the chaotic system offers good performance in signal enlargement. Second, the properties of aperiodic long-term and deterministic behavior are extended using “Circuit Implementation of Synchronized Chaos with Applications to Communications” [23], edited by Kevin M. Cuomo and Alan V. Oppenheim. The spectrum of the chaotic system is wide enough to be applied in signal encryption [24]. We can use this phenomenon to produce chaotic mapping for the analysis, as the spectra of the signals have no limits.

After feature extraction, we choose extension theory as our identification method. Compared to other identification methods, extension theory does not require tedious parameter setting or a significant amount of calculations. These factors save not only computational resources but also computing time [13]. Compared to classical mathematical sets and fuzzy sets, extension sets have a wider area that can describe the degree of belonging. That is, extension sets can describe the degree of belonging in more detail. Taking fuzzy sets [25,26] and extension sets [27] as examples, the former only define the degree of belonging from 0 to 1, while the extension theory extends this to −∞ to +∞. Expanding the degree of belonging can offer a more detailed description, which has the potential to decrease classification errors. For this reason, we choose extension theory for this study. To summarize, we apply a method that combines a fractional order Chen–Lee chaotic system and extension theory to offer a shorter detection time and greater diagnosis rate. Finally, we present five feature extraction methods that have relatively high correct rates.

After the diagnostic procedure is determined, we will explain how to combine vibration signals with chaotic systems, feature extraction, and extension theory. The original vibration signal is a one-dimensional signal that is delayed and fed into the Chen–Lee chaotic system (x, y, z) in three dimensions as a preprocessing step. Then, the five proposed feature formulas are applied to the x, y, and z vectors, which is known as feature extraction. Finally, the diagnostic value calculated from feature extraction serves as a reference to classify and assess the accuracy of the input signal as either a normal state, ball fault, inner race fault, or outer race fault using extension theory.

The organization of this paper is described as follows. In Section 2, we discuss how to obtain the data source and perform data preprocessing. In Section 3, we present the advantages of using chaotic systems, explain how to process chaotic mapping, and demonstrate a phase diagram under various loading and failure states. In Section 4, inspired by the phase diagram, we propose five different feature extraction methods and apply them using the five formulae. In Section 5, we introduce extension theory and define the classic domain and joint field. In Section 6 and Section 7, we present the classification results and summarize the diagnosis conclusions.

## 2. Data Processing

### 2.1. Data Resource

**Figure 1:**
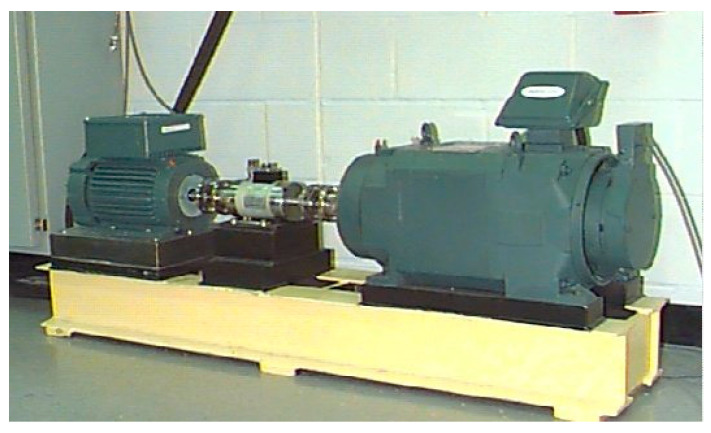
Ball bearing experiment platform.

The data used for simulation were obtained from Case Western Reserve University Bearing Data Center of the United States [28], which provides ball bearing test data for normal and faulty bearings. The test stand, depicted in Figure 1, is composed of a 2 hp motor on the left, a torque transducer/encoder in the center, and a dynamometer on the right. The motor shaft is supported by the test bearings, each subjected to a single point of failure (SPOF) using electro-discharge machining (EDM) with diameters of 7, 14, 21, 28, and 40 mils, as specified in the fault specifications, which also indicate fault depths of 0.011 inches. The defective bearings were reinstalled into the test motor. Then, we recorded vibration data for motor loads with different levels of horsepower. Finally, data were collected for normal bearings, single-point drive ends, and fan end defects. Then, we saved the results in MATLAB format. The test specifications are shown in Table 1. The fan end was the last section of the data resource and had a lower sampling rate; only the normal bearing and the SPOF drive end were used. Four states were provided on the inner raceway, rolling element (i.e., ball), outer raceway, and normal types.

### 2.2. Data Processing

Data were collected at 12,000 samples/second and 48,000 samples/second for the drive-end bearing experiments. As a result, the lengths of the data may be different.

For the simulation, we chose a sampling frequency of 48 k for the drive-end bearing experiments. However, the data length of 0 HP was different from that of other HPs. The length of the former was 240,000 samples and that of the latter was 480,000 samples. The 0 to 48,000th samples were not taken into consideration because the motor start-up caused one second transient state.

Additionally, data for SPOF with 0.014 inch on the inner race for 0 HP and 1 HP needed to be extended in order to obtain the same data length as other types of faults. After processing, the data were separated into two parts. One part was used for data training, and the other was used to verify the results shown in Table 2.

## 3. Chaotic Master–Slave System

### 3.1. Chaos Theory

The chaotic phenomenon results from the fact that objects continue replicating the state of motion in previous stages with some certain rules, which leads to unpredictable random effects. Therefore, a chaotic system is often used to discuss the behaviors that cannot be explained in dynamic systems with a single data relationship but can be predicted by a comprehensive data relationship. The chaotic system has some notable properties. First, the motion trajectory of the system is extremely sensitive to the initial value. Additionally, the chaotic system is a nonlinear system with randomness, indicating that the system has some potential principles that govern the system’s evolution, which can be predicted in a certain category and regarded as an important factor affecting the operation of the system. In this study, the above properties are used to identify the ball bearings in different states.

### 3.2. Chaotic Mapping

**Figure 2:**
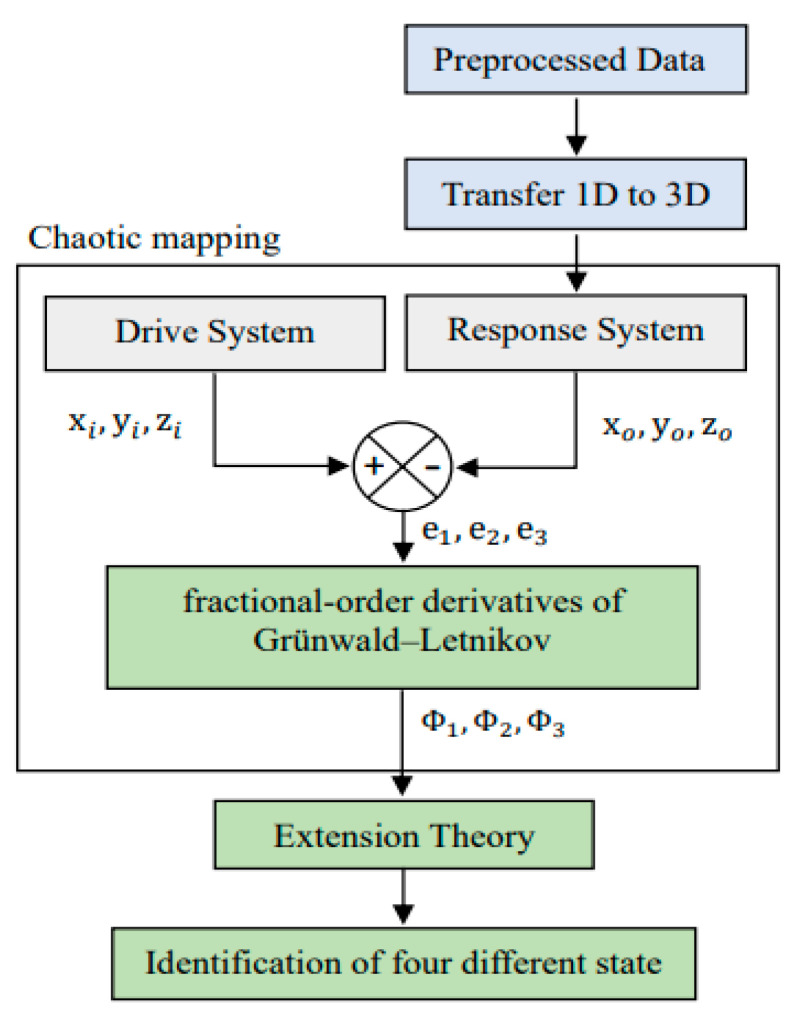
The procedure for converting original data into dynamic errors via chaotic mapping for ball bearing condition identification.

As Figure 2 shows, the ball bearing data are first preprocessed and transformed from one- to three-dimensional forms. Next, the data are entered into the chaotic mapping system, including a drive system *x_i_*, *y_i_*, *z_i_* and a response system *x_o_*, *y_o_*, *z_o_*, where *x_i_*, *y_i_*, *z_i_* are the coordinates of a fixed point and *x_o_*, *y_o_*, *z_o_* are the three-dimensional data of the ball bearing. Both systems are calculated by the Chen–Lee chaotic equations [29], represented as Equations (1) and (2).

(1) $$\left\{\begin{array}{c} x_{i}=-y_{i}z_{i}+ax_{i}\\ y_{i}=x_{i}z_{i}+by_{i}\\ z_{i}=\frac{1}{3}x_{i}y_{i}+cz_{i} \end{array}\right.$$

(2) $$\left\{\begin{array}{c} x_{o}=-y_{o}z_{o}+ax_{o}+u_{1}\\ y_{o}=x_{o}z_{o}+by_{o}+u_{2}\\ z_{o}=\frac{1}{3}x_{o}y_{o}+cz_{o}+u_{3} \end{array}\right..$$

Here, the response is subtracted from the driving system to form a set of dynamic errors, that is, e_1_, e_2_, e_3_. By using the fractional order derivatives of Grünwald–Letnikov [14], as in (3), an extra variable α can be obtained, and the new dynamic errors Φ_1_, Φ_2_, Φ_3_ are entered into the extension theory to identify different states of the ball bearing system.

(3) $$D_{e}^{\alpha }e^{m}\approx \frac{\Gamma \left(m+1\right)}{\Gamma \left(m+1-\alpha \right)}e^{m-\alpha }$$

**Figure 3:**
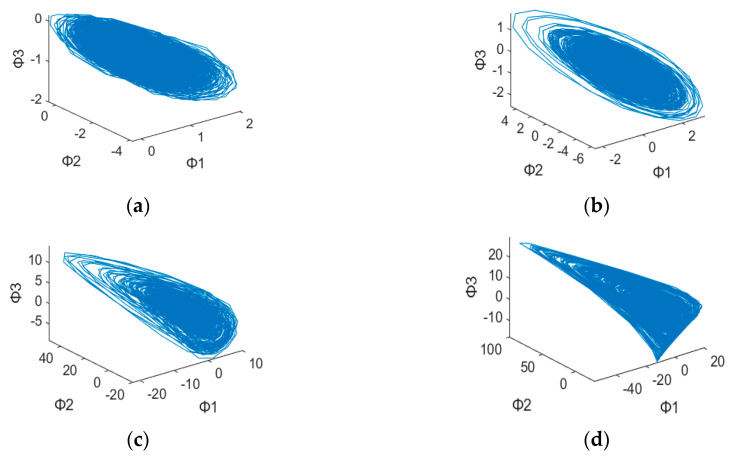
3D dynamic error phase diagram of a ball bearing under testing, with α = 0, HP = 0, and SPOF diameter = 0.007 in. (**a**) Normal state. (**b**) Ball fault. (**c**) Inner race fault. (**d**) Outer race fault.

**Figure 4:**
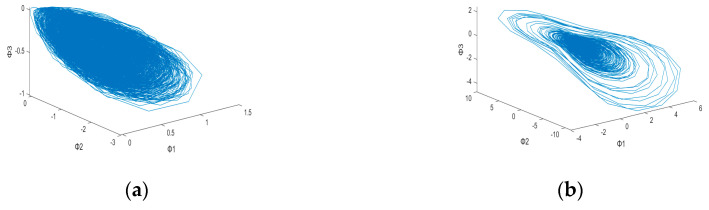

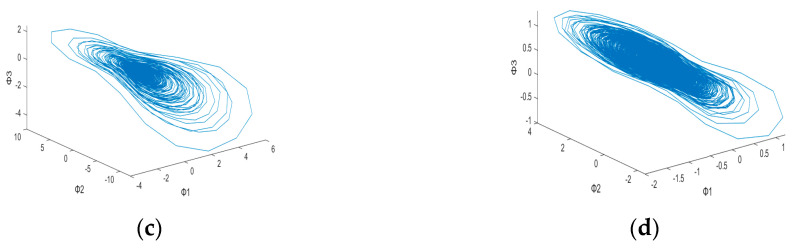
3D dynamic error phase diagram of a ball bearing under testing, with α = 0.3, HP = 0, and SPOF diameter = 0.007 in. (**a**) Normal state. (**b**) Ball fault. (**c**) Inner race fault. (**d**) Outer race fault.

**Figure 5:**
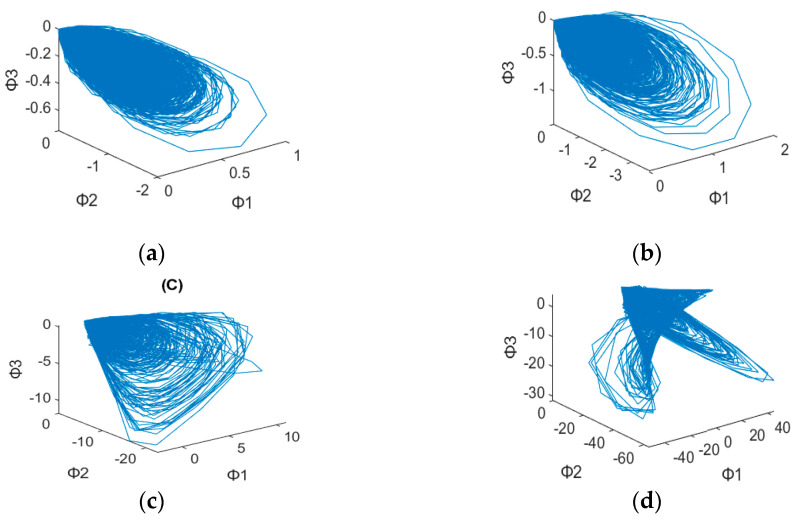
3D dynamic error phase diagram of ball bearing under testing, with α = 0.6, HP = 0, and SPOF diameter = 0.007 in. (**a**) Normal state. (**b**) Ball fault. (**c**) Inner race fault. (**d**) Outer race fault.

**Figure 6:**
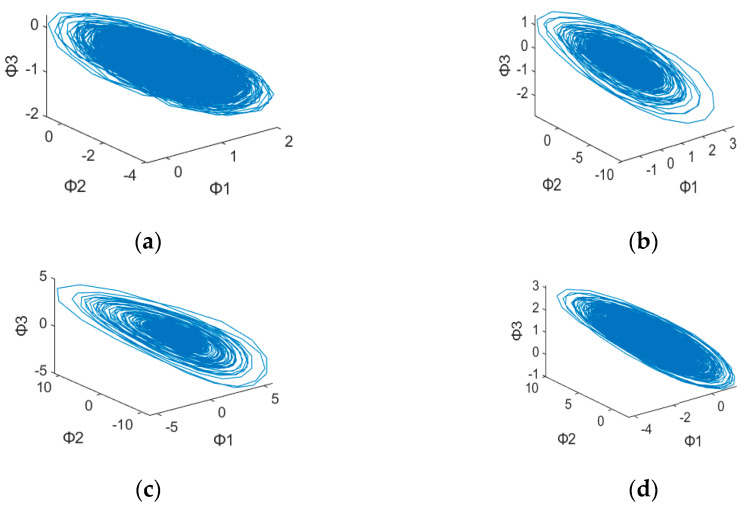
3D dynamic error phase diagram of ball bearing under testing, with α = 0, HP = 0, and SPOF diameter = 0.014 in. (**a**) Normal state. (**b**) Ball fault. (**c**) Inner race fault. (**d**) Outer race fault.

**Figure 7:**
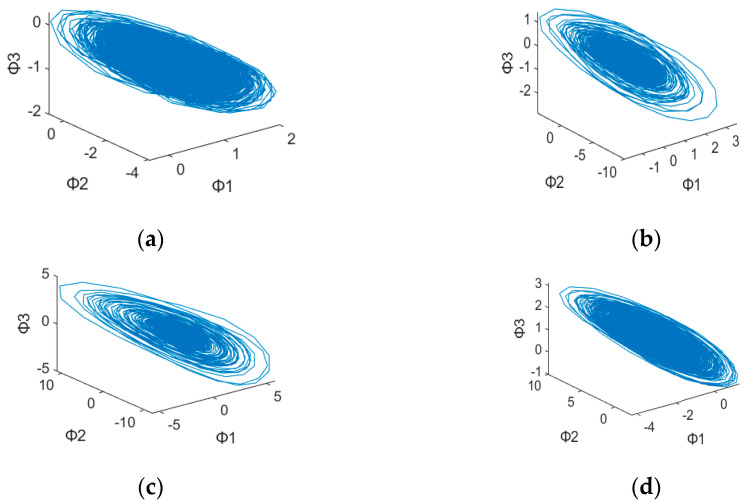
3D dynamic error phase diagram of ball bearing under testing, with α = 0.3, HP = 0, and SPOF diameter = 0.014 in. (**a**) Normal state. (**b**) Ball fault. (**c**) Inner race fault. (**d**) Outer race fault.

**Figure 8:**
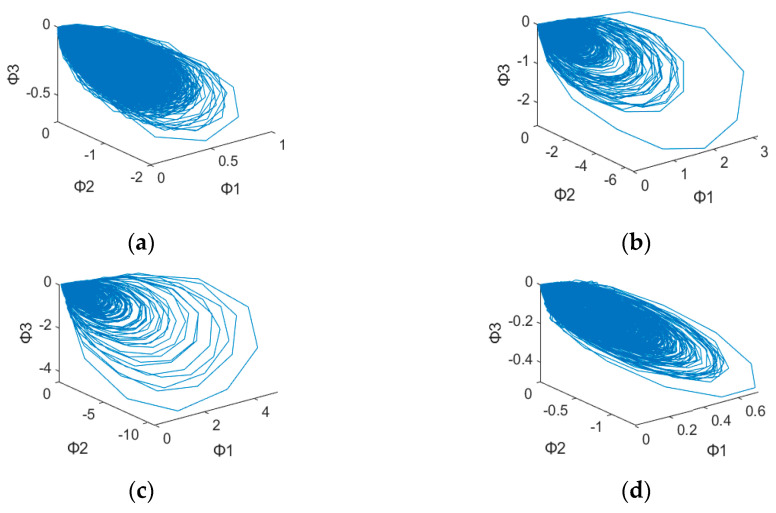
3D dynamic error phase diagram of ball bearing under testing, with α = 0.6, HP = 0, and SPOF diameter = 0.014 in. (**a**) Normal state. (**b**) Ball fault. (**c**) Inner race fault. (**d**) Outer race fault.

After plotting the 3D dynamic error phase diagram with different values of α, the potential features maps are illustrated in Figure 3, Figure 4, Figure 5, Figure 6, Figure 7 and Figure 8 with α = 0, 0.3 and 0.6, which is also listed in Table 3. For α = 0, in Figure 3a,b, these are similar in both numerical range and shape, while Figure 3c,d have similar shapes, making them difficult to differentiate. Figure 6 has a significant overlap in the range of all four fault states, and the graphs and distribution densities are also similar, making analysis difficult. Both graph parameters are α = 0, so they are not ideal. For α = 0.6, Figure 5 and Figure 8 also have wide numerical ranges, but the distribution densities are very close to the origin, so they are also not suitable for use with parameters that are α = 0.6. Finally, For α = 0.3, in Figure 4b,c, these have similar shapes and ranges, but there is enough difference in the numerical range of Figure 4a,d to allow for classification. In Figure 7, there is a significant difference in the numerical distribution range, allowing for classification. According to the analysis mentioned above, an α value of 0.3 is decided, which is more suitable for diagnosis of bearing status.

## 4. Feature Extraction—Five Feature Extraction Methods for Performance Investigation

In order to identify the four different conditions of the ball bearing system, we must perform feature extraction. Feature extraction is a process used to simplify the initial data that allows data to be allocated to a more manageable group to facilitate learning and maintain data integrity. Sometimes, feature extraction can even offer a more complete interpretation of the initial data. The following are the five feature extraction methods used in this article:

(4) $$\sqrt{{\left(\Phi_{x}-{\left(\Phi_{x}\right)}_{max}\right)}^{2}+{\left(\Phi_{y}-{\left(\Phi_{y}\right)}_{max}\right)}^{2}}$$

(5) $${\left(\Phi_{x}\right)}_{max}/{\left(\Phi_{x}\right)}_{min}$$

(6) $$\left({\left(\Phi_{x}\right)}_{max}-{\left(\Phi_{x}\right)}_{min}\right)\times \left({\left(\Phi_{y}\right)}_{max}-{\left(\Phi_{y}\right)}_{min}\right)$$

(7) $$\sqrt{{\left({\left(\Phi_{x}\right)}_{ave}\right)}^{2}+{\left({\left(\Phi_{y}\right)}_{ave}\right)}^{2}}$$

(8) $$\sqrt{{\left(\Phi_{y}-{\left(\Phi_{y}\right)}_{ave}\right)}^{2}}$$

After using Euclid’s distance between 2D dynamic errors and their maxima, the first method takes the maximum values as the characteristics. From the observations of the 2D phase diagram, we found that the graphs exhibit a ribbon distribution with different sizes. Therefore, the maximum values of *x* and *y* are deducted from the test points, and the Euclidean distance is taken to make the result close to the long side of the distribution. Then, we take the maximum value as the eigenvalue.

The second method uses only the *x* direction of dynamic errors, which is the ratio of maximum and minimum value. There are obvious differences between the maximum and minimum values of the four states in the *x* and *y* directions when observing the size of the 2D phase diagram. The difference in the *x* direction is more obvious, so we take the ratio of the maximum and minimum values in the *x* direction as one of the methods.

In the third method, we subtract the minimum values from the dynamic errors in the *x* and *y* directions and then multiply those values. According to the 2D phase diagram, the area of each graphic is different. It is suitable to use this property to identify the vibration signals. Since the area of the graphic is too difficult to calculate, we use the area of the rectangle, which represents the maximum and minimum area of the graphic.

Next, we use Euclid’s distance between the average point and origin of the 2D dynamic errors. Each average point of the vibration signals is separated in the 2D phase diagram, so the distribution of these points is useful for identification. We then calculate the value of Euclid’s distance between the average point and origin and use it as one of the characteristics for this study.

The last method is almost identical to the first but uses only the y direction, rather than 2D dynamic errors. The range of distribution for the characteristic values is known as the classical domain, which is included in the joint field. By observing the 2D phase diagram, the value of Φ_2_ under different error states is shown to be quite different. Therefore, the feature extraction method in method 5 mainly focuses on Φ_2_. We extract the maximum value of the distance between the Φ_2_ value of each point and the average value of Φ_2_ as the feature value and apply this method to this study.

## 5. Extension Theory

**Figure 9:**
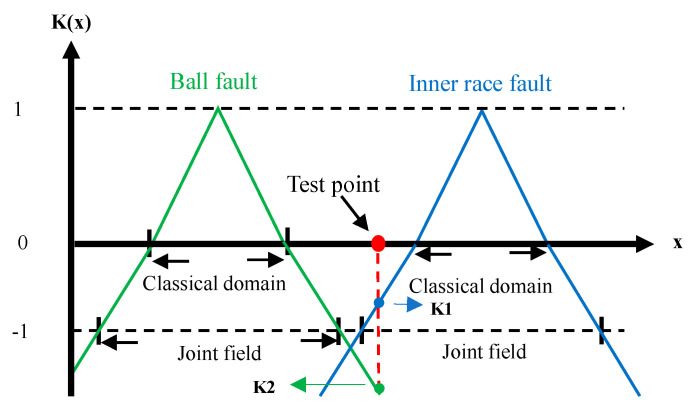
Illustration of a correlation function.

Generally, extension theory [30,31,32,33] is a method that helps to determine how related an object is to the feature by using an extension set and correlation function. Figure 9 presents a schematic of an extension set and correlation function. Briefly speaking, Figure 9 illustrates two triangles with different colors, and the horizontal and vertical axes represent the eigenvalues and correlation function, respectively. Additionally, assume that the green and blue triangles represent the correlation functions of the ball fault and inner race fault, respectively. Next, we compare K1 and K2, which represent the values of the correlation function shown in Figure 9, to determine which fault state the test point belongs to. Here, the test point is closer to the inner race fault, so the value of the correlation function is larger, i.e., K1 > K2. Thus, we classify this test point as the inner race fault. Eventually, by applying extension theory, we can compare the magnitude of each correlation function to determine which error state an observation belongs to.

## 6. Experimental Results

We conducted the multiple steps of this research using MATLAB. There are five main steps for feature extraction and testing.

- Use pre-processed training and testing data to generate dynamic errors in the Chen–Lee chaotic mapping system.
- Calculate the characteristics of dynamic errors based on the variety of the methods of feature extraction.
- Set the classical domain and joint field by continuously training the characteristics.
- Calculate the correlation functions of the different testing conditions with the testing characteristics, classical domain, and joint field in extension theory.
- Compare the values of the correlation functions to determine the testing data (i.e., the vibration signal of the normal state, the ball fault state, the inner race fault state, and the outer race fault state).

In this research, we use five methods for feature extraction and three sets, each of which is handled by two methods. The weight of each method in the sets is 0.5. Table 4, Table 5, Table 6 and Table 7 show the classical domain and joint field of the second sets using different HP and 0.007 in SPOF diameters. In the table, the “X” symbol represents the third method, and the “Y” symbol represents the fourth method.

For the testing results, the accuracy of 0, 1, 2, and 3 HP is expressed in Table 8, Table 9, Table 10 and Table 11, respectively. The average accuracy is the average of the normal state, ball fault state, inner race fault state, and outer race fault state accuracy for the same SPOF and the same HP. This value represents the accuracy under a particular condition. In Table 12, the total average accuracy refers to the average accuracy in all conditions.

As shown in Table 12, the highest average accuracy in feature extraction was achieved by the second set. For feature extraction, this set used Euclid’s distance of the average area of a rectangle, determined by the maximum and minimum values in the 2D dynamic error phase diagram as characteristics. These two characteristics represent, respectively, the position and area information of the graphics. When a difficult condition, such as the 0.014 in SPOF diameter testing data, needs to be determined, the second set offers better effects because it provides the two different properties of dynamic errors.

## 7. Conclusions

This study focused on the effects of different feature extraction methods in the diagnosis of ball bearing vibration signals. After generating dynamic errors with the chaotic system, we assessed five feature extraction methods to calculate the classical domains and joint fields and then tested the accuracy of identification with extension theory under different conditions. According to the results, to obtain the highest accuracy ratio, it is necessary to analyze the properties of the dynamic errors in the phase diagram and to determine the appropriate characteristics.

Five feature extraction methods were proposed in this study, of which two were chosen as the basis for Methods 1 to 3. Method 1 used Equations (4) and (5), Method 2 used Equations (6) and (7), and Method 3 used Equations (7) and (8) as the two features X and Y for extension theory classification. The accuracy obtained from experiments using these three methods is presented in Table 8, Table 9, Table 10, Table 11 and Table 12, with Table 12 displaying the average accuracy of the three methods. The accuracy rates for Method 1, Method 2, and Method 3 were found to be 87.8%, 94.4%, and 84.8%, respectively. After comparing the results, we determined that Method 2 achieved the best and most stable classification results among the three methods. This paper also proposed a preprocessing step that utilizes a chaotic mapping system. Five different feature extraction equations were then used to develop three methods that utilized the two main features of X and Y. Finally, extension theory was employed for classification. Ultimately, our results show that the properties of feature extraction are the key factor needed to find the most accurate method for determining the ball bearing vibration signal. In the future, we will use the architecture in this study to develop improved methods for feature extraction with improved accuracy.

## Figures and Tables

**Table 1:** Data Information.

Parameters	Types
Sampling rate	48 k (Hz)
Motor load	0/1/2/3 (Hp)
SPOF diameter	0.007/0.014/0.021 (in)
SPOF depth	0.011 (in)
Type of fault	Normal/Inner Race Fault/Ball Fault/Outer Race Fault

**Table 2:** Training Data and Testing Data.

	Training Data	Testing Data
Motor load 0 Hp with different diameters of faults	48,000–144,000th	144,001–240,000th
Motor load 1/2/3 Hp with different diameters of faults	48,000–264,000th	264,001–480,000th

**Table 3:** 3D Dynamic Error Phase Diagram of Ball Bearing Under Testing.

Figure\State	SPOF	α
Figure 3	0.007 in	α = 0
Figure 4	0.007 in	α = 0.3
Figure 5	0.007 in	α = 0.6
Figure 6	0.014 in	α = 0
Figure 7	0.014 in	α = 0.3
Figure 8	0.014 in	α = 0.6

**Table 4:** Classical Domain and Joint Field When SPOF Diameter = 0.007 in, HP = 0.

State	Classical Domain	Joint Field
Normal	X <3.336 4.811> Y <1.071 1.090>	X <2.968 5.179> Y <1.066 1.095>
Ball fault	X <1106 1464> Y <1.204 1.275>	X <1017 1554> Y <1.186 1.293>
Inner race fault	X <16.69 30.97> Y <1.028 1.062>	X <13.12 34.55> Y <1.020 1.070>
Outer race fault	X <7383 9604> Y <1.574 1.649>	X <6828 10,158> Y <1.555 1.669>

**Table 5:** Classical Domain and Joint Field When SPOF Diameter = 0.007 in, HP = 1.

State	Classical Domain	Joint Field
Normal	X <3.336 4.811> Y <1.071 1.090>	X <2.968 5.179> Y <1.066 1.095>
Ball fault	X <1106 1464> Y <1.204 1.275>	X <1017 1554> Y <1.186 1.293>
Inner race fault	X <16.69 30.97> Y <1.028 1.062>	X <13.12 34.55> Y <1.020 1.070>
Outer race fault	X <7383 9604> Y <1.574 1.649>	X <6828 10,158> Y <1.555 1.669>

**Table 6:** Classical Domain and Joint Field When SPOF Diameter = 0.007 in, HP = 2.

State	Classical Domain	Joint Field
Normal	X <2.756 6.106> Y <1.066 1.101>	X <1.919 6.944> Y <1.058 1.109>
Ball fault	X <222.0 287.0> Y <1.277 1.330>	X <205.8 303.3> Y <1.264 1.343>
Inner race fault	X <13.50 24.10> Y <1.125 1.184>	X <10.85 26.75> Y <1.109 1.199>
Outer race fault	X <4568 6023> Y <1.598 1.690>	X <4204 6387> Y <1.575 1.713>

**Table 7:** Classical Domain and Joint Field When SPOF Diameter = 0.007 in, HP = 3.

State	Classical Domain	Joint Field
Normal	X <3.000 4.538> Y <1.059 1.116>	X <2.615 4.922> Y <1.045 1.130>
Ball fault	X <183.5 237.6> Y <1.303 1.349>	X <170.0 251.1> Y <1.291 1.361>
Inner race fault	X <12.42 27.98> Y <1.132 1.184>	X <8.531 31.87> Y <1.118 1.197>
Outer race fault	X <4976 6454> Y <1.602 1.670>	X <4606 6824> Y <1.585 1.687>

**Table 8:** Accuracy When HP = 0.

Method	Method 1			Method 2			Method 3		
SPOF Diameter (10^−3^ in)	7	14	21	7	14	21	7	14	21
Normal (%)	100	100	100	100	83.9	98.2	100	80.2	92.4
Ball fault (%)	100	100	100	100	100	100	100	100	100
Inner race fault (%)	100	59.9	100	100	88.4	100	100	65	100
Outer race fault (%)	100	0	100	100	100	100	100	100	100
Average (%)	100	64.9	100	100	93.1	99.6	100	86.3	98.1

**Table 9:** Accuracy When HP = 1.

Method	Method 1			Method 2			Method 3		
SPOF Diameter (10^−3^ in)	7	14	21	7	14	21	7	14	21
Normal (%)	100	100	100	100	100	100	100	90.9	100
Ball fault (%)	100	89.9	90.4	100	68.8	100	100	88.7	100
Inner race fault (%)	100	91.9	84.2	100	100	100	100	91.1	100
Outer race fault (%)	100	100	100	100	71.4	100	100	92.7	100
Average (%)	100	95.5	93.6	100	85.1	100	100	90.8	100

**Table 10:** Accuracy When HP = 2.

Method	Method 1			Method 2			Method 3		
SPOF Diameter (10^−3^ in)	7	14	21	7	14	21	7	14	21
Normal (%)	100	100	100	100	100	100	100	100	100
Ball fault (%)	100	100	100	100	71.3	100	100	100	100
Inner race fault (%)	100	0	100	100	88.6	100	100	0	0
Outer race fault (%)	100	0	100	100	51.3	100	100	0	100
Average (%)	100	50	100	100	77.8	100	100	50	75

**Table 11:** Accuracy When HP = 3.

Method	Method 1			Method 2			Method 3		
SPOF Diameter (10^−3^ in)	7	14	21	7	14	21	7	14	21
Normal (%)	100	100	100	100	87.6	88	100	76.3	85.7
Ball fault (%)	100	100	100	100	98.5	100	100	100	100
Inner race fault (%)	100	0	100	100	69.7	98.7	100	0	0
Outer race fault (%)	100	0	96.6	100	68.2	100	100	6.4	100
Average (%)	100	50	99.1	100	81	96.6	100	46.5	71.4

**Table 12:** Total Average Accuracy.

Method	Method 1	Method 2	Method 3
Total Average Accuracy (%)	87.8	94.4	84.8

## Data Availability

No new data were created in this paper.
